# 4-Hydroxyphenylacetate 3-Hydroxylase (4HPA3H): A Vigorous Monooxygenase for Versatile *O*-Hydroxylation Applications in the Biosynthesis of Phenolic Derivatives

**DOI:** 10.3390/ijms25021222

**Published:** 2024-01-19

**Authors:** Ping Sun, Shuping Xu, Yuan Tian, Pengcheng Chen, Dan Wu, Pu Zheng

**Affiliations:** The Key Laboratory of Industrial Biotechnology, Ministry of Education, School of Biotechnology, Jiangnan University, Wuxi 214122, China; 7200201021@stu.jiangnan.edu.cn (P.S.); 6230202015@stu.jiangnan.edu.cn (Y.T.); chenpengcheng@jiangnan.edu.cn (P.C.); wudan@jiangnan.edu.cn (D.W.)

**Keywords:** 4-hydroxyphenylacetate 3-hydroxylase (4HPA3H), two-component flavin-dependent monooxygenase, *o*-hydroxylation, plant polyhydroxyphenols, plant-derived P450-alternative monooxygenases

## Abstract

4-Hydroxyphenylacetate 3-hydroxylase (4HPA3H) is a long-known class of two-component flavin-dependent monooxygenases from bacteria, including an oxygenase component (EC 1.14.14.9) and a reductase component (EC 1.5.1.36), with the latter being accountable for delivering the cofactor (reduced flavin) essential for *o*-hydroxylation. 4HPA3H has a broad substrate spectrum involved in key biological processes, including cellular catabolism, detoxification, and the biosynthesis of bioactive molecules. Additionally, it specifically hydroxylates the *o*-position of the C4 position of the benzene ring in phenolic compounds, generating high-value polyhydroxyphenols. As a non-P450 *o*-hydroxylase, 4HPA3H offers a viable alternative for the de novo synthesis of valuable natural products. The enzyme holds the potential to replace plant-derived P450s in the *o*-hydroxylation of plant polyphenols, addressing the current significant challenge in engineering specific microbial strains with P450s. This review summarizes the source distribution, structural properties, and mechanism of 4HPA3Hs and their application in the biosynthesis of natural products in recent years. The potential industrial applications and prospects of 4HPA3H biocatalysts are also presented.

## 1. Introduction

Catechols and trihydroxyphenols, including caffeic acid [1], piceatannol [2], and salvianic acid A [3], are natural bioactive molecules with remarkable antioxidant, antibacterial, and anti-proliferative activities that are important chemical and pharmaceutical intermediates with great significance in the pharmaceutical, cosmetic, food, and polymer material industries [4]. Bioconversion has been a green and sustainable alternative employed in the production of polyphenols. This approach not only reduces the cultivation period of the plant that produced them, but also enhances the composite efficacy by tailoring novel enzymes for further derivatization. However, there is still a distance from practical industrial applications the complexity of enzymes derived from plants. Therefore, establishing an efficient alternative hydroxylation method is of great practical value. Among them, the application of the synthesis pathway of polyphenols containing 4-hydroxyphenylacetate 3-hydroxylase is attractive.

4-Hydroxyphenylacetate 3-hydroxylase (4HPA3H) is a class of two-component flavin-dependent monooxygenases consisting of an oxygenase component (EC 1.14.14.9) and a reductase component (EC 1.5.1.36). It can specifically and catalytically introduce hydroxyl groups to the *o*-position of phenol analogs to synthesize new phenolic compounds. Therefore, 4HPA3H has great potential in the degradation of environmental pollutants and the synthesis of valuable plant polyphenols [5,6].

Initially, 4-hydroxyphenylacetate 3-hydroxylase was discovered to participate in the catabolism of various organic compounds, including *p*-hydroxyphenylacetate (4-HPA), aniline, tyrosine, and aromatic amines [7,8]. It plays a pivotal role in the microbial degradation of aromatic compounds by executing the primary hydroxylation step [9]. During the investigation of organic compounds’ degradation by microorganisms such as *Pseudomonas* and *Penicillium*, it was found that they could grow on a gel plate using 4-HPA as the only carbon source. Adachi [10] et al. identified 3,4-dihydroxyphenylacetic acid (3,4-DHA) when studying the metabolic 4-HPA of *Pseudomonas ovalis*. They then named the involved enzyme *p*-Hydroxyphenylacetate hydroxylase and speculated that it comprised at least two components. *Acinetobacter*, *Pseudomonas putida* [11], and *Escherichia coli* [12] (except for K-12 and NCTC 5928) were successively proved to have enzymes that oxidize 4-HPA to 3,4-DHA. In 1992, two components of *p*-hydroxyphenyl 3-hydroxylase from *P. putida* [13] were purified for the first time, and the mechanism was subsequently analyzed in 1994 [14]. In 1993, Prieto [5] et al. characterized a broad-spectrum aromatic hydroxylase utilizing 4-HPA in *E. coli* W (ATCC 11105), classifying it as 4-hydroxyphenylacetate hydroxylase and naming the key gene *hpa*B. In 1994, 4-hydroxyphenylacetate 3-hydroxylase was sequenced on the genome of *E. coli* W, becoming the first member of the two-component aromatic hydroxylase family [14]. At this time, both 4-HPA hydroxylases from *P. putida* and *Acinetobacter* were considered to belong to the same class as those from *E. coli* W, i.e., 4-hydroxyphenylacetate 3-hydroxylases. The enzyme derived from *E. coli* W was reported as a FAD-dependent two-component monooxygenase [15]. In addition, Gibello [16,17] et al. found 4-hydroxyphenylacetate 3-hydroxylase derived from *Klebsiella pneumoniae* could utilize not only 4-HPA but also other substrates, including 3-hydroxyphenylacetic acid, 4-hydroxybenzoic acid, L-dihydroxyphenylalanine, L-tyrosine, and catechol.

Plant polyphenols have the potential to benefit human health and can be further divided into flavonoid compounds (e.g., flavonoids, isoflavones, and anthocyanins) and non-flavonoid compounds (e.g., phenolic acids, lignans, stilbenes, and tannins) based on their structures [18]. Polyphenols, as bioactive compounds, possess antioxidant, anti-inflammatory, and anti-cancer properties, contributing to the prevention and treatment of chronic diseases. These effects are achieved through interactions with the gut microbiota and phytochemicals, thereby enhancing cardiac and cognitive health [19]. The sustainable recovery of polyphenols from fruit waste enables the development of functional foods, pharmaceuticals, and food additives, promoting human health and environmental protection [20]. The antioxidant properties of phenolic acids are significantly affected by the type of spacer between carboxylic acids and aromatic rings [21]. Additionally, the number and location of hydroxyl groups in phenolic compounds are directly related to their antioxidant capacity [22]. It is generally believed that phenolic compounds with a greater number of phenolic hydroxyl groups (e.g., dihydric phenols or trihydric phenols) show stronger antioxidant capacities. In the 21st century, natural products with monophenol structures in plants have been hydroxylated to biologically active diphenol or polyphenol compounds using 4HPA3Hs.

The C-H oxyfunctionalization of phenolic compounds remains a distinct challenge in chemosynthesis. Plant extraction is limited by raw materials, environment, time, and quantity. Moreover, the low content and instability of polyphenols from plants make the extraction tricky [23]. The synthesis of dihydric phenols and trihydric phenols in plants usually requires P450 hydroxylases [24]. However, some plant-derived P450s with complex intrinsic catalytic mechanisms, along with low expression levels and diminished electron transfer efficiency, significantly impede the promotion and application of polyphenols. Notably, 4HPA3Hs can be easily heterologously expressed and have the same function for *o*-hydroxylation executed by certain P450s. They can cost-effectively convert monophenols into high-value polyphenols in vitro, which is highly suitable for replacing unique P450s in plants, such as *p*-coumaric acid 3-hydroxylase (C3H), flavonoid 3-hydroxylase (F3H), ferulate 5-hydroxylase (F5H), and flavonoid 3’, 5’-hydroxylase (F3’5’H) [24]. Moreover, 4HPA3Hs provide a viable strategy for the direct synthesis of several phenol analogs with medicinal value, including flavonoids and alkaloid analogs.

This review describes the catalytic properties, structural functions, and biotechnological applications of 4-hydroxyphenylacetate 3-hydroxylases. It highlights the potential application of 4HPA3Hs as alternatives to plant P450s with *o*-hydroxylation functionality in the synthesis of plant polyphenols. It also outlines the strategies that can be applied to increase the productivity of natural products catalyzed by 4HPA3Hs and points out potential future directions for the development of 4-hydroxyphenylacetate 3-hydroxylases.

## 2. Characteristics of 4-Hydroxyphenylacetate 3-Hydroxylases

### 2.1. Classifications of 4-Hydroxyphenylacetate 3-Hydroxylases

4-Hydroxyphenylacetate 3-hydroxylase plays a critical role in the catabolism of natural or synthetic aromatic compounds and the biosynthesis of plant polyphenols [25,26]. It utilizes flavin as a cofactor and NAD(P)H as an electron donor. Flavin-dependent monooxygenases have been classified into groups A-H according to the structural properties, sequence motifs, and electron donor and oxygenation reactions [26,27]. 4HPA3H is categorized as a member of group D for its typical acyl-CoA dehydrogenase domain [26,28]. Other members in group D include 4-nitrophenol 2,4-monooxygenase; 4-chlorophenol monooxygenase; 2,4,5-trichlorophenol 4,2-monooxygenase; 2,4,6-trichlorophenol 4,6-monooxygenase; phenazine monooxygenase; naphthocyclinone monooxygenase; and indole monooxygenase [26,29,30]. The various 4HPA3Hs exhibit different reductase and oxygenase components, which are summarized in Table 1 [31].

4-Hydroxyphenylacetate 3-hydroxylases have been discovered in numerous microorganisms capable of degrading organic compounds, such as *A. baumannii* [7,42], *E. coli* [14], *Pseudomonas putida* [41], *Sulfolobustokodaii*, *Thermus thermophilus* [32], and *Klebsiella pneumoniae* [16] (Table 1). Variations in 4HPA3Hs from various microorganisms lead to differences in their catalytic properties and applications. For example, the optimal temperature and pH for 4HPA3Hs from *Geobacillus thermodenitrificans* NG80-2 are 60 °C and pH 9, respectively [39]. It has been demonstrated that 4HPA3Hs can hydroxylate various non-natural phenolic compounds, such as phenol, chlorophenol, styrene, and *p*-nitrophenol [17]. Furthermore, they can be employed in the oxidation of natural phenolic compounds to enhance their antioxidant properties. 4HPA3Hs are a versatile class of phenol hydroxylases capable of specifically hydroxylating phenolic-structure analogs to produce catechols.

**Figure 1:**
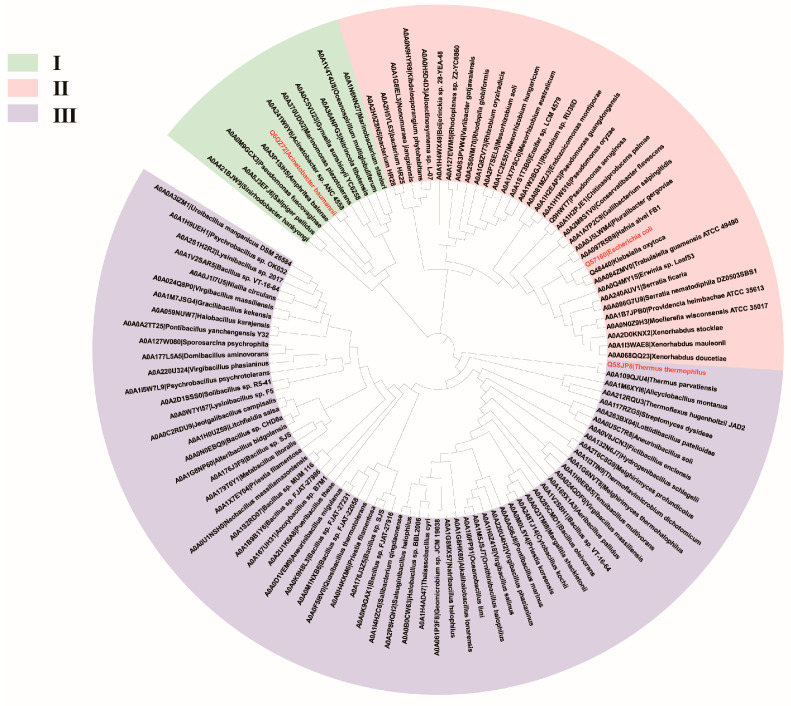
The phylogenetic tree of HpaBs. Amino acid sequences were obtained from the UniProt database, with the accession numbers and species shown. MEGA7 was used to align multiple protein sequences using the neighbor-joining method and construct the phylogenetic tree. The tree comprising 112 member proteins was visualized using iTOL. HpaBs from *A. baumannii* (I), *E. coli* (II), and *T. thermophilus* (III) are marked in red.

In the hydroxylation reaction catalyzed by 4HPA3Hs, the oxygenase component is the key part executing the hydroxylation in the presence of the substrate and cofactor FAD, which is supplied by the reductase component. The oxygenase component is commonly referred to as HpaB, except for that from *A. baumannii*, which is known as C2. Though many sequences of HpaBs have been identified, only three crystal structures of HpaBs have been obtained, isolated from *A. baumannii* (*Ab*HpaB, Unprot ID: Q6Q272), *T. thermophilus* (*Tt*HpaB, Unprot ID: Q5SJP8), and *E. coli* (*Ec*HpaB, Unprot ID: Q57160). Then, three sequences were used as probes to search for more potential HpaBs in the UniProt database by BLASTP (amino acid sequence identity ≥ 30%, coverage ≥ 80%, and e-value ≤ 1 × 10^−10^). As shown in Figure 1, 112 sequences of HpaBs from a variety of bacteria were classified into three main branches (I, II, and III). *Ab*HpaB in branch I displayed a low sequence similarity with *Tt*HpaB(III) and *Ec*HpaB(II), at 20.51% and 15.19%, respectively. *Tt*HpaB in branch III and *Ec*HpaB in branch II had a higher sequence similarity at 30.72%. Branch II had a closer phylogenetic relationship with III compared to I (in fact, branch I was quite distant from branches II and III, while the phylogenetic tree was created by ignoring the actual distance). Therefore, 4HPA3Hs containing HpaBs in branch I are defined as FMN/FAD-dependent monooxygenases, while others are FAD-dependent monooxygenases. HpaBs in the same branch are generally from the same or closely related species and tend to exhibit similar catalytic properties. For instance, *Pa*HpaB shares a 73% sequence similarity with *Ec*HpaB, and both of them have broad substrate spectrum, including *p*-cumaric acid, resveratrol, tyrosol, and tyramine [36,43]. Most of HpaBs in branch III are from *Bacillus* and tend to exhibit a higher thermostability.

### 2.2. Structural Features of 4HPA3H

#### 2.2.1. Structural Features of 4HPA3Hs’ Oxygenase Components

**Figure 2:**
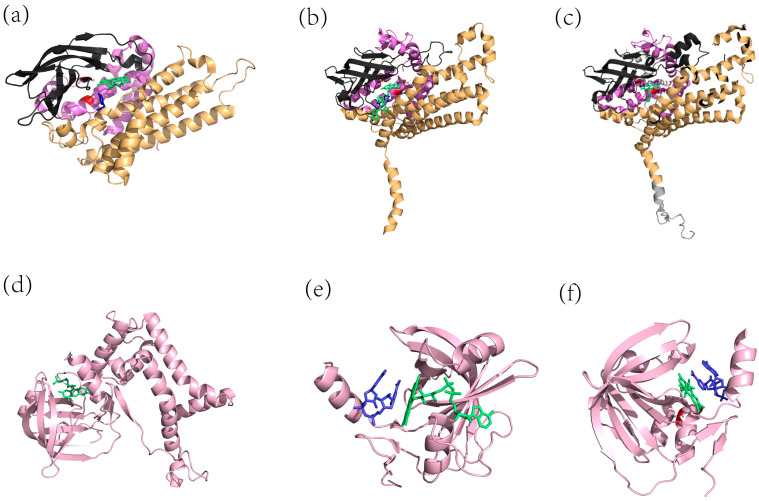
Crystal structures of HpaB and HpaC monomers (**a**) *Ab*HpaB (PDB ID: 2JBT, key residues in red: His120 and Ser146); (**b**) *Tt*HpaB (PDB ID: 2YYJ, key residues in red: Arg100, Tyr104, and His142); and (**c**) *Ec*HpaB (PDB ID:6QYI, key residues in red: Arg113, Tyr117, and His155). N-terminal domain is purple, the middle domain is black, the C-terminal domain is orange, FAD or FMN is green, and 4-HPA is blue. Key residues for substrate binding are highlighted in red. (**d**) *Ab*HpaC (PDB ID:5ZC2); (**e**) *Tt*HpaC (PDB ID: 2ED4); (**f**) *St*HpaC (PDB ID: 2D37, key residue in red: Phe79). FAD or FMN is green, and NADH is blue. Key residues for substrate binding are highlighted in red.

To date, the three crystal structures of HpaBs from *A. baumannii*, *E. coli*, and *T. thermophilus* have been obtained, and the details are provided in Table 2. They are homotetramers with low sequence similarities but have highly similar structures. The monomer of each homotetramer can be approximately divided into three structural domains: the N-terminal domain, the intermediate domain, and the C-terminal domain. The C-terminal domain of the monomer is crucial for maintaining the stability of the tetrameric structure. It is evident that the monomers have an α-helix tail at the C-terminal, except for *Ab*HpaB (Figure 2a). *Ec*HpaB has an additional “extension” compared to *Tt*HpaB (Figure 2b,c).

The oxygenase component derived from *A. baumannii* (*Ab*HpaB; UniProt ID: Q6Q272) is a homotetramer with a monomer molecular weight of 50 kDa, which prefers FMNH^−^ than FADH^−^ as a cofactor [7]. The monomer of *Ab*HpaB comprises an N-terminal domain (residues 24–143), a β-sheet domain (144–237), and a C-terminal domain (238–422). The crystal structure of *Ab*HpaB has a cavity that encapsulates and stabilizes the C4a-hydroperoxyflavin intermediate [44,45]. The residues Tyr207 and Phe216 are involved in the binding of 4-HPA. Mutations in Phe266 resulted in a significant decrease in C4a-hydropterin production rates [46], because the gatekeeper residue Phe266 controlled oxygen entry into the *Ab*HpaB active site. Thotsaporn [47] et al. demonstrated that the hydroxyl side chain of Ser-171 interacting with the flavin N5 is essential for the stability of C4a-hydroperoxy-FMN. His396 was shown to be important for the formation of the C4a-hydroperoxyflavin intermediate within the active site of *Ab*HpaB, but did not participate in H_2_O_2_ elimination. His120 and Ser146 are essential for substrate binding and efficient hydroxylation. Thermostability studies [48] showed that *Ab*HpaB was highly stable, retaining activity after incubation at 30, 35, and 40 °C for 24 h. Kinetic studies [49] indicated that *Ab*HpaB bound tightly to reduced FMN (*K_d_* 1.2 ± 0.2 µM). The C2:FMNH^−^ preferred to form a stable C(4a)-hydroperoxy-FMN intermediate with O_2_ (*k* = 1.1 ± 0.1 × 10^6^ M^−1^s^−1^) rather than with the substrate 4-HPA. The hydroxylation reaction occurred in the ternary complex with the substrate, forming a C2:C(4a)-hydroxy:FMN:DHPA complex with a rate constant of 20 s^−1^. *Ab*HpaB accepted a narrower range of substrates than *Ec*HpaB [35].

*Tt*HpaB (UniProt ID: Q5SJP8) derived from *T. thermophilus* is an (α_2_)_2_ tetrameric protein in which the C-terminus of *Tt*HpaB consists of a long α-helix that wraps around both dimers. The conserved amino acids R100-Y104-H142 are crucial for substrate binding. The crystal structure analysis of *Tt*HpaB (Figure 2b) revealed that flavin binding and dissociation are accompanied by conformational changes between the β5-β6 loop and the β8-β9 loop, leading to the formation of partial substrate binding sites (Ser-197 and Thr-198) [32]. The loop between β8 and β9 had a conformational change and shielded the active site from the solvent upon binding with 4-HPA. Arg100 located near the putative oxygen binding site may be related to the formation and stabilization of the C4-hydroperoxyflavin intermediate. This enzyme exhibited a broad substrate range, including 4-HPA, chlorophenol, styrene, phenol, *p*-nitrophenol, nitrotriacetic acid, and L-tyrosine. The *Tt*HpaB-FAD-HPA ternary complex structure was relatively easy to obtain compared to *Ec*HpaB [32,34,35].

Since 2019, the structure and function of *Ec*HpaB have been characterized [34,35]. *Ec*HpaB has an (α_2_)_2_ tetrameric structure, with each monomer comprising three structural domains: an N-terminal α-helix domain (1–151), a core domain with a β-barrel structure (152–284), and a C-terminal α-helix domain (285–520). Although the *Ec*HpaB and *Tt*HpaB structures are highly similar, their sequence identity is low at 30.72%. The ternary complex of *Ec*HpaB bound to FAD and HPA has not yet been obtained, and only the crystal structure of the *Ec*HpaB-FAD complex (Figure 2c) is available.

The residues directly involved in FAD binding are R164, Y461, H155, I157, V158, and T196 (isoalloxazine ring) [35]. The Arg113, Tyr117, and His155 residues conserved in *Ec*HpaB are vital for substrate binding. The β32-β33 loop (207–217) of *Ec*HpaB is responsible for substrate specificity, exhibiting remarkable plasticity and high tolerance to extensive mutation. The flexible loop that allows substrates to access and stabilize at the active site is a key factor for enzyme multifunctionality. The loop is essential for substrate binding and specificity and has been demonstrated to have significant conformational changes upon FAD and ligand binding in *Tt*HpaB [34]. S210 and A211 may participate in stabilizing the 4-HPA tail, determining substrate selectivity [50]. The mutants V158G, I157A, I157G, and I157S no longer possess hydroxylase activity, whereas S210A and S210Q mutants retain hydroxylase activity [35]. *Ec*HpaB exhibits difficulty in oxidizing the substrates once the phenyl ring is dual-hydroxylated, except for 3,4-DHA [35].

Among the three enzymes from different origins, only *Ab*HpaB demonstrates a preference for the cofactor FMN, while others are FAD-dependent monooxygenases. The region associated with flavin specificity, referred to as the “flavin-binding loop”, is only found in HpaBs from FAD-dependent monooxygenases and is absent in *Ab*HpaB. The “flavin-binding loop” region containing an arginine and a glutamine is conserved in FAD-specific monooxygenases. Owing to the disordered nature of the loop in the absence of a flavin cofactor, this region cannot be observed in the X-ray structures of the apoenzyme (devoid of a cofactor). *Ec*HpaB is the only oxygenase component whose crystal structure of the ternary complex has not been obtained. Moreover, it possesses a broader substrate range compared to those of *At*HpaB and *Tt*HpaB. It is concluded that *Ec*HpaB has a larger substrate-binding pocket capable of accommodating bulkier phenolic compounds.

#### 2.2.2. Structural Features of 4HPA3Hs’ Reductase Components

As the smaller subunit in 4HPA3Hs, the reductase component is a homodimer with the primary function of providing the preferred reduced flavin to the oxygenase component. To date, the crystal structures of the reductase components from *A. baumannii*, *T. thermophilus*, and *Sulfolobus tokodaii* Strain 7 have been reported (Figure 2d–f, Table 3). Modifications at the C-terminus have been found to affect the flavin reduction and dissociation. For instance, Wang [51] et al. observed a severe impairment in activity when an S-tag was added to the C-terminus of *Ec*HpaC.

The reductase component derived from *A. baumannii* (*Ab*HpaC) is commonly referred to in the majority of reports as C1. The reaction mechanism of 4HPA3H from *A. baumannii* shares similarities with that of bacterial luciferases. The presence of the substrate promotes NADH hydroxylation, indicating that *Ab*HpaC has a 4-HPA substrate-binding sites. *Ab*HpaC is the first aromatic flavone hydroxylase utilizing reduced FMN. The N-terminal of *Ab*HpaC has the binding sites for flavin and NADH, while the C-terminal may be responsible for the stimulation of NADH oxidation. The crystal structures revealed that *Ab*HpaC consists of an N-terminal flavin reductase domain and a C-terminal MarR domain [52]. A unique feature of *Ab*HpaC is the presence of a regulatory site binding 4-HPA and is stimulated by the substrate. The binding of 4-HPA to the C-terminal MarR domain of *Ab*HpaC induced structural changes, thereby relieving self-inhibition [52]. The binding of 4-HPA to the enzyme enhanced flavin reduction capability by at least 30-fold [53], and several phenolic compounds can stimulate flavin reduction. This phenomenon is not common among flavin-dependent monooxygenases, except for *Ab*HpaC. Kinetic studies suggested that reduced flavin (FMNH^-^) can diffuse from *Ab*HpaC to *Ab*HpaB without any protein–protein interaction and bound tightly to *Ab*HpaB before binding to O_2_ [54]. The rate-limiting step of the hydroxylation reaction is the dissociation of *Ab*HpaC with FMNH^−^. The regulatory mechanism of *Ab*HpaC in the flavin reduction step shows that the activation is in the control of the C-terminal domain. The C-terminal domain acts as a self-inhibitory domain, can activate flavin reduction by conformational changes, and releases the reduced flavin when bound to 4-HPA [52,55].

The *T. thermophilus*-derived reductase component *Tt*HpaC consists of 149 residues, forming a central groove for binding FAD and NADH. The isoalloxazine ring of FAD and the nicotinamide ring of NAD face each other on the surface. A comparison of the reductase enzymes of the two-component flavin-diffusible monooxygenases (TC-FDMs) family revealed that *Tt*HpaC prefers NADH over NADPH. The crystal structure analysis of *Tt*HpaC indicated no conformational changes occurred upon FAD binding. The preference of *Tt*HpaC for FAD is related to the interaction between the AMP moiety of FAD and *Tt*HpaC’s non-conserved loop (Gly83-Gly94). *Tt*HpaC demonstrates a stronger affinity for FAD, with a *K_m_* value of 8.9 µM for FAD and 36.8 µM for FMN [33].

Okai and colleagues [40] obtained the crystal structures of the short-chain flavin reductase *St*HpaC from *Sulfolobus tokodaii* Strain 7 in three states: apo (without NAD(P)), NAD-bound, and NADP-bound. These structures demonstrate that *St*HpaC exists as a homodimer, exhibiting a preference for FMN and NADH. NADH and NADPH were found to bind to *St*HpaC at the same position but with opposite orientations. The Phe79 residue interacted with FMN and was conserved among homologous proteins.

### 2.3. Catalytic Mechanism of 4-Hydroxyphenylacetate 3-Hydroxylase

**Figure 3:**
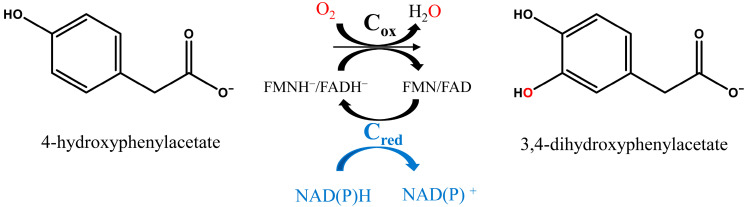
Reaction scheme of 4-hydroxyphenylacetate 3-hydroxylases (C_red_: the reductase component; C_ox_: the oxygenase component).

4HPA3H specifically catalyzes the *o*-hydroxylation of phenolic compounds. In the hydroxylation reaction of 4-HPA (Figure 3), the reductase component (C_red_) provides reduced flavin using NADH and FMN/FAD as substrates and then the oxygenase component (C_ox_) applies reduced flavin and O_2_ (dioxygen) to obtain the dihydric phenol (3,4-DHA). During the oxidation reaction, one oxygen atom of the dioxygen atoms is introduced into the hydroxylation product, while the other one is reduced to H_2_O and eliminated.

**Figure 4:**
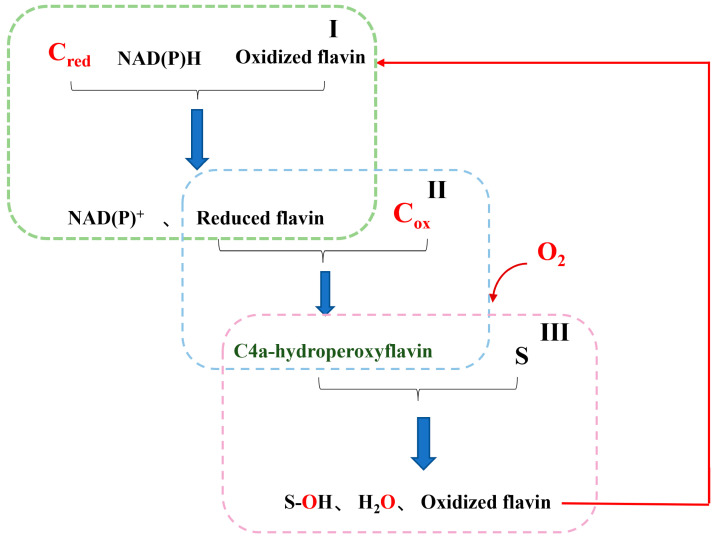
Flow chart of *o*-hydroxylation by 4-hydroxyphenylacetate 3-hydroxylases.

The catalytic reaction process of 4-hydroxyphenylacetate 3-hydroxylase generally consists of three steps (Figure 4): In Step I, the reduced flavin is provided by the reductase component (C_red_) using NAD(P)H as an electron donor. In Step II, the supplied reduced flavin binds to the oxygenase component (C_ox_) via free diffusion, and then reacts with molecular oxygen to form the stable C4a-hydroperoxyflavin intermediate [7]. In Step III, the intermediate interacts with the substrate (S) to form hydroxylation products with the release of water and oxidized flavin that proceeds to the next hydroxylation cycle [56,57].

**Figure 5:**
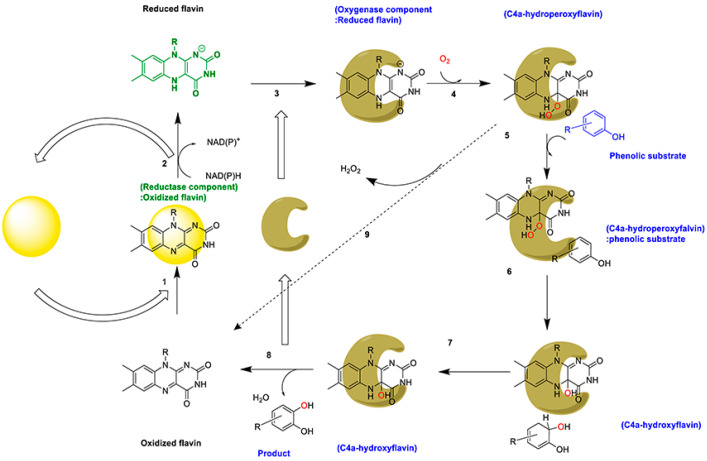
Overall reaction of 4-hydroxyphenylacetate 3-hydroxylases [28,31].

The reductase component executes the reduction half-reaction, followed by the oxygenase component carrying out the oxidation half-reaction (Figure 5). During the reduction half-reaction, the following occurs (1–2 steps): The oxidized flavin binds to the reductase component to form a (reductase component: oxidized flavin) complex (step 1). Subsequently, flavin reduction occurs in the presence of NAD(P)H (step 2). The oxidation half-reaction (steps 3–8) begins with the reduced flavin binding to the oxygenase component, synthesizing the (oxygenase component: reduced flavin) complex (step 3). This complex interacts with O_2_, generating a stable C4-hydroperoxyflavin intermediate (step 4).

The C4-hydroperoxyflavin intermediate then binds to phenolic compounds to synthesize the (C4-hydroperoxyflavin:phenolic substrate) complex (step 5). The C4-hydroxyflavin intermediate and the perhydroxylated product are then released (step 6). A hydroxylated product, oxidized flavin, and H_2_O are further produced (steps 7–8). In the absence of substrates, the stable C4-hydroperoxyflavin intermediate decomposes autolytically to H_2_O and oxidized flavin (step 9). The oxidized flavin enters the next hydroxylation cycle [31].

During the entire catalytic reaction process, flavin, as a cofactor, remains in a state of in situ regeneration. The hydroxylation reaction catalyzed by 4-hydroxyphenylacetate 3-hydroxylase relies on the continuous consumption of NAD(P)H in the presence of the preferred flavin. The reduced flavin acts as one of the substrates for the oxygenase component, ultimately leading to substrate hydroxylation mediated by dioxygen. Therefore, the hydroxylation reaction requires the provision of sufficient NAD(P)H, O_2_ and appropriate oxidized/reduced flavin. Interestingly, Deng [35] et al. reported that an organometallic complex [Rh(bpy)Cp*(H_2_O)Cl]Cl (Cp*:1,2,3,4,5-pentamethylcyclopentadiene, bpy: 2,2′-bipyridyl) and formate could be used for FAD reduction instead of reductase components.

As described in Section 2.1, 4HPA3Hs have diverse origins, with significant sequence variations and different properties among them. 4HPA3Hs exhibit a broad substrate spectrum. There is some variation in substrate specificity and stability among 4HPA3Hs from different sources. For example, despite a high sequence similarity of 73% between *Pa*HpaB and *Ec*HpaB, *Pa*HpaB can utilize ferulic acid, while *Ec*HpaB cannot. Recently, most studies have highlighted the thermostability of *Ac*HpaBC and its mutants [48,58], but detailed information on the thermostability of 4HPA3Hs from alternative sources is limited.

The hydroxylation efficiency of 4HPA3H mainly depends on its oxygenase component. The kinetic parameters of oxygenase components (HpaBs) are shown in Table 4. It is evident that HpaBs from *E. coli* are the most widely utilized for the synthesis of natural polyhydroxyphenols. *Ec*HpaB has a high affinity (*K_m_* = 137.6 ± 21.0 μM) for small substrates, such as 4-HPA, tyrosine, and *p*-coumaric acid, while demonstrating a lower affinity for large-sized molecules, like naringenin and umbelliferone [34]. *Ro*HpaB has a higher activity than that of *Ec*HpaB, *Kp*HpaB, and *Pp*HpaB in bulky substrates (e.g., naringenin) [59]. In 2014, Lin and Yan [60] et al. successfully hydroxylated umbelliferone, resveratrol, and naringenin (in a low activity) by overexpressing *E. coli*-derived 4HPA3H. This process resulted in the production of esculetin (2.7 g/L), piceatannol (1.2 g/L), and eriodictyol, respectively. Herrmann [61] et al. showed that HpaBC from *E. coli* had 100% conversion of *p*-coumaric acid and rheosmin, whereas only 39% conversion of *p*-hydroxybenzoic acid. Zhou [62] et al. found that *Kp*HpaB had a higher hydroxylation activity towards *p*-coumaric acid than *Pp*HpaB, *Ec*HpaB, and their mutants (*K_m_* = 725.19 ± 6.82 μM), which facilitated the construction of an engineered strain for FA production. As described above, 4HPA3Hs are emerging as popular *o*-hydroxylases in the development of high-value natural products.

## 3. Biosynthesis of Phenolic Derivatives by 4HPA3Hs

4HPA3Hs have a broad substrate specificity and can be used in the biosynthesis of natural products, including monocyclic phenols and polycyclic phenols. Table 5 lists some dihydric phenols obtained by various monooxygenases. 4HPA3H appears to be more appealing for various biological applications. Most of the *o*-hydroxylation of aromatic compounds is catalyzed by P450s in plants. Details are shown in Section 3.1 and Section 3.2.

P450s have sophisticated mechanisms requiring a specific redox partner protein and several rich cofactors during the *o*-hydroxylation process, but usually lower catalytic efficiencies. There is a research gap regarding the expression of the high activity of P450s. Therefore, the discovery of alternative enzymes of plant P450s is important for the biosynthesis of high-value natural products. Enzymes that effectively *o*-hydroxylate phenolic compounds have attracted great interest. Compared to plant-derived P450s with low expression and inefficient electron transport efficiency in prokaryotic hosts, non-P450 hydroxylases from bacteria show great superiority in constructing cell factories. Notably, the aforementioned polyhydroxyphenols in Table 5 can also be biosynthesized by 4HPA3Hs. It further shows that 4HPA3Hs have a great potential to establish a platform for the biocatalytic synthesis of natural products. In this review, we primarily highlight the merits of 4HPA3Hs in enzymatically synthesizing a range of representative polyhydroxyphenols.

### 3.1. Hydroxylation of Monocyclic Phenols

*p*-Hydroxycinnamic acid derivatives are typical monocyclic phenols in lignin biosynthesis, including *p*-coumaric acid, ferulic acid, and caffeic acid. These compounds exhibit pharmacological value due to their antioxidant, anti-ultraviolet, and anticancer activities. They are widely used as monomers and functional additives in the synthesis of customized food, cosmetics, pharmaceuticals, and plastics.

Caffeic acid (3,4-dihydroxycinnamic acid) is a plant-derived phenolic compound synthesized in plants by a membrane-associated cytochrome P450, known as *p*-coumaric acid 3-hydroxylase (C3H). In 2011, Kim [79] et al. successfully expressed the *Arabidopsis* C3H with in *E. coli* through N-terminal truncation. However, the application of plant C3H in prokaryotic hosts was challenging. In 2013, *Arabidopsis* C3H was first functionally expressed in the *cyanobacterium Synechocystis* PCC 680374. Additionally, it was commonly characterized with bioactive in *Saccharomyces cerevisiae* [65,80]. However, the relatively low activity of C3H still limits the application in caffeic acid synthesis. In 2012, Lin and Yan [81] et al. completely converted 100 mg/L of *p*-coumaric acid to caffeic acid within 3 h by expressing endogenous 4HPA3H in *E. coli*. The mutant CYP199A2_F185L was co-expressed with putidaredoxin reductase (Pdr) and palustrisredoxin (Pux) in *E. coli* and successfully produced 2.8 g/L caffeic acid after 24 h [67]. Huang [82] et al. overexpressed the 4HPA3Hs from *E. coli* MG1655 (*Ec*HpaBC) and *T. thermophilus* HB8 (*Tt*HpaBC) in *E. coli* BW25113 with the plasmid pZE12-luc. The latter was better for the synthesis of caffeic acid from *p*-coumaric acid (3.5 g/L), and the yield was 3.82 g/L at 24 h. Furuya [36] et al. first overexpressed the 4HPA3H from *Pseudomonas aeruginosa* PAO1 (*Pa*HpaBC) in *E. coli* BL21 (DE3) and obtained 10.2 g/L caffeic acid after 24 h by the repeated addition of *p*-coumaric acid. In 2016, Jones [83] et al. overexpressed endogenous 4HPA3H in *E. coli* to hydroxylate *p*-coumaric acid and obtained caffeic acid at the yield of 3.5 g/L. In 2019, Liu [84] et al. combined *Pa*HpaB and *Se*HpaC in *S. cerevisiae* and achieved the highest yield of caffeic acid at 289.4 ± 4.6 mg·L^−1^, which was about 43 times higher than that of the strain containing *Ec*HpaBC. In 2022, Zhang [66] et al. synthesized 18.74 g/L (0.85 g/(L·OD_600_)) caffeic acid at 6 h by whole-cell catalysis with the engineered *E. coli* overexpressing its endogenous 4HPA3H. The conversion rate of *p*-coumaric acid reached 78.81%, marking the highest level of caffeic acid production reported to date.

4HPA3H derived from *E. coli* can not only synthesize caffeic acid but also convert tyrosol to hydroxytyrosol [81]. Hydroxytyrosol, the most abundant dihydroxyphenol in virgin olive oil, exhibits diverse pharmacological activities that are suitable for applications in the food and health industries. In 2001, Espín [68] synthesized hydroxytyrosol using mushroom tyrosinase as a biocatalyst and ascorbic acid as a reductant. In 2013, Orenes-Piñero [85] et al. hydroxylated tyrosol to the corresponding hydroxytyrosol using the 4HPA3H from *G. thermoglucosidasius*. Furuya [36] et al. showed that *Pa*HpaBC catalyzed the hydroxylation of tyrosol with a 66% conversion of the substrate (10 mM) within 1 h. Moreover, Yao [86] et al. overexpressed *Ec*HpaBC-coupled D-lactate dehydrogenase for the de novo synthesis of salvianic acid A, which resulted in a final yield of 7.1 g/L. In 2020, Zeng [87] et al. cascaded *Ec*HpaBC and L-DOPA decarboxylase (DODC) to convert 5 mM of L-tyrosine to L-dopamine with over 90% conversion, and then 32.35 mM hydroxytyrosol was obtained from 50 mM L-tyrosine. Furthermore, 4HPA3Hs were used as tyrosine hydroxylases, resulting in 95% conversion of tyrosine to L-DOPA [88].

5-Hydroxyferulic acid(5-OHFA) is a dihydric phenol found in a variety of fruits [89], vegetables [90], and cereals [91]. 5-Hydroxyferulic acid has much higher antioxidant and hydrophilic properties than monophenols (ferulic acid) and has a great potential effect in pharmaceuticals, cosmetics, and food additives [92,93]. In plants, ferulate 5-hydroxylase (F5H) synthesizes 5-hydroxyferulic acid by adding a hydroxyl group to the C5 position of the ferulic acid phenyl ring [94,95]. In 1999, F5H was expressed in the *S. cerevisiae* WAT11 [69] and INVSc2 [96], catalyzing the conversion from FA to 5-OHFA. It was first reported that *Pa*HpaBC can catalyze the hydroxylation of cinnamic acid derivatives, such as caffeic acid as well as ferulic acid and coniferaldehyde, which correspond, respectively, to reactions catalyzed by CYP98A and CYP84A enzymes in plants. And the conversion of ferulic acid (10 mM) was 4.1% and 53% at 1 h and 12 h, respectively [36]. In 2022, Herrmann [61] et al. engineered a mutant Y301I of *Ec*HpaBC to recognize and hydroxylate ferulic acid, achieving a conversion rate of 45% for 200 µM ferulic acid at 16 h. In that paper, *Pa*HpaBC seemed to have a larger substrate-binding pocket than *Ec*HpaBC and was able to hydroxylate the C5 position with C3 as a pre-existing substituent.

4HPA3Hs can introduce a hydroxyl group not only at the C3 position but also at the C5 position within the phenol structure. 4HPA3Hs from *A. baumannii* [48], *Klebsiella pneumoniae* [17], and *E. coli* [35] were also reported to catalyze 3,4-DHA and some other dihydric phenols to generate corresponding trihydric phenols. This further indicates that certain 4HPA3Hs could go on to hydroxylate the C5 position to synthesize dihydric phenols or trihydric phenols, either subsequent to the hydroxylation of the C3 position in the monophenol or based on pre-existing substituents at the C3 position. For example, 3,4-DHA and caffeic acid, ferulic acid, and other such substances with hydroxyl or methoxyl substitutions at both the C4 and C3 positions can still be *o*-hydroxylated by 4HPA3Hs, suggesting that 4HPA3Hs is an excellent *o*-hydroxylase.

Otherwise, 4HPA3Hs can synthesize aminophenol derivatives, which are precursors of some pharmacologically active compounds, such as 3-hydroxy-4-aminophenylacetic acid (3-OH-4-APA). *Ab*HpaBC was able to catalyze the *o*-hydroxylation reaction of 4-aminophenylacetic acid to generate 3-OH-4-APA [97], and the variant was able to achieve *o*-hydroxylation of tyramine and octopamine [98]. It also implies that the substrate scope of 4-hydroxyphenylacetate 3-hydroxylase extends beyond phenolics, encompassing a broader class of structural analogs characterized by a hydroxyl or amino group at the C4 position of the benzene ring, which further expands its field of application.

### 3.2. Hydroxylation of Polycyclic Phenols

The structural composition of numerous natural products with considerable medicinal value, including anti-tumor, anti-inflammatory, and anti-cancer properties, surpasses that of simple monocyclic aromatic compounds. In fact, more valuable natural substances often feature intricate polyaromatic ring systems, such as polycyclic aromatic compounds exemplified by stilbenes and flavonoids [99]. 4HPA3Hs specifically recognize the phenolic moiety within phenolic compounds and demonstrate a high tolerance for bulky molecules, enabling them to hydroxylate more complex aromatic compounds.

Piceatannol is an excellent tyrosine kinase inhibitor and inhibitor of cancer cell proliferation and growth. CYP1A2 [100], CYP1B12, P450BM3 (CYP102A1), and the P450BM3 mutant F87A [101,102] all have been reported to be involved in the biotransformation of trans-resveratrol to piceatannol, but the yield was low. In 2012, Lee [70] et al. showed that the tyrosinase from *Streptomyces avermitilis* MA4680 regionally and selectively hydroxylated trans-resveratrol in the presence of catechol and inhibited the further oxidation of the product by tyrosinase. In 2014, *Pa*HpaBC was used to produce 23 mM (5.2 g·L^−1^) piceatannol from resveratrol through whole-cell catalysis [71], changing the previous notion that 4HPA3Hs were catalytically active exclusively for monocyclic aromatic compounds. This suggests that 4HPA3Hs may have potential applicability for other oxidative functions involving high-value polycyclic compounds.

3’-hydroxylation is an important step of the biosynthetic pathway of flavonoids [24] and is generally facilitated by cytochrome P450-dependent monooxygenation [24,103]. In recent years, the employment of 4HPA3Hs for the synthesis of 3’-hydroxylated flavonoids has garnered growing interest in the scientific community. Naringin, dihydrokaempferol, kaempferol, and apigenin [24] are the prevalent polycyclic aromatic compounds in the downstream flavonoid pathway. They can be synthesized by F3’H or F3’5’H (Table 5) to yield the corresponding hydroxylated flavonoids, but the conversion rate in plants is low [24,72,104,105]. The P450BM3 mutant M13 was able to hydroxylate naringenin and yielded 13.5 mg/L of eriodictyol at 48 h with a substrate conversion rate of 49.81% [74]. In 2020, Gao [72] et al. overexpressed F’3H and cytochrome P450 reductase (CPR) from *Silybum marianum* in *S. cerevisiae* and obtained 3.3 g/L eriodictyol with a 62% conversion rate. The functional activity of *Ec*HpaBC to convert naringenin and afzelechin to their corresponding 3’-hydroxylated flavonoids was determined with the titer of eriodictyol 62.7 ± 2.7 mg/L [83]. In 2021, Wang [51] et al. overexpressed endogenous 4HPA3H in *E. coli* and obtained 46.84 ± 2.85 mg/L eriodictyol with a conversion rate of 57.67 ± 3.36%. In 2022, Wu and co-workers [73] first overcame the conversion bottleneck in eriodictyol synthesis by employing *E. coli*-derived 4HPA3Hs as a substitute for F’3H in *Corynebacterium glutamicum*, ultimately achieving a yield of 14.10 mg/L. In another research, 4HPA3H from *Rhodococcus opacus* (*Ro*HpaBC) was used to convert naringenin, apigenin, and kaempferol into eriodictyol, luteolin, and quercetin, respectively [51]. Notably, the Y215A mutant exhibited a conversion rate of up to 77% for naringenin. Additionally, some studies have focused on the engineered *Ec*HpaBC based on its structure or sequence to improve its activity towards naringenin [59,61]. The CYP102A1 mutant M10 (R47L/F87V/L188Q) was used for the 3-hydroxylation of phloretin, producing 3.1 mM 3-hydroxyphloretin, which is a typical dihydrochalcone [76]. In 2023, Xu [64] et al. first proved that *Pa*HpaBC also *o*-hydroxylated phloretin and worked better than M10.

Equol, a soy isoflavone metabolite, has been reported to be metabolized to 6- or 3′-hydroxyequol and 6, 3′-di hydroxyequol by P450s in rat or human liver microsomes [106]. Mushroom tyrosinase was found to convert equol to 3′-hydroxyequol in the study of the inhibitory effect of equol [77]. *Ec*HpaB and the variant T292A were proved to hydroxylate the C6 position of equol to 6-hydroxyequol [107]. Both HpaB_ro-3_ derived from *Rhodococcus opacus* B4 and HpaB_pl-1_ derived from *Photorhabdus luminescens* can hydroxylate equol, but at different hydroxylation sites [78,108]. In 2019, Hashimoto [108] et al. discovered that HpaB_pl-1_ could hydroxylate equol to 6-hydroxyequol, while HpaB_ro-3_ hydroxylated the C3′ position of equol, yielding 1.06 g/L (S)-3′-hydroxyequol. When both HpaB_ro-3_ and HpaB_pl-1_ worked together in the hydroxylation of equol, the substrate was eventually converted into 6,3′-hydroxyequol [78].

In addition to the previously mentioned phenols, *Ec*HpaBC, which has been extensively investigated, can also hydroxylate polycyclic aromatic compounds, including resveratrol, umbelliferone, and 2-hydroxycarbazole (an alkaloid analog) [61]. It implies that 4-hydroxyphenylacetate 3-hydroxylase is an unrivaled, highly efficient *ortho*-hydroxylase for substrates ranging from cinnamic acid derivatives and flavonoid derivatives to alkaloid derivatives, essentially covering the majority of natural products. As a result, it emerges as a particularly appealing alternative to certain plant-derived P450s for *o*-hydroxylation applications of plant phenols.

**Figure 6:**
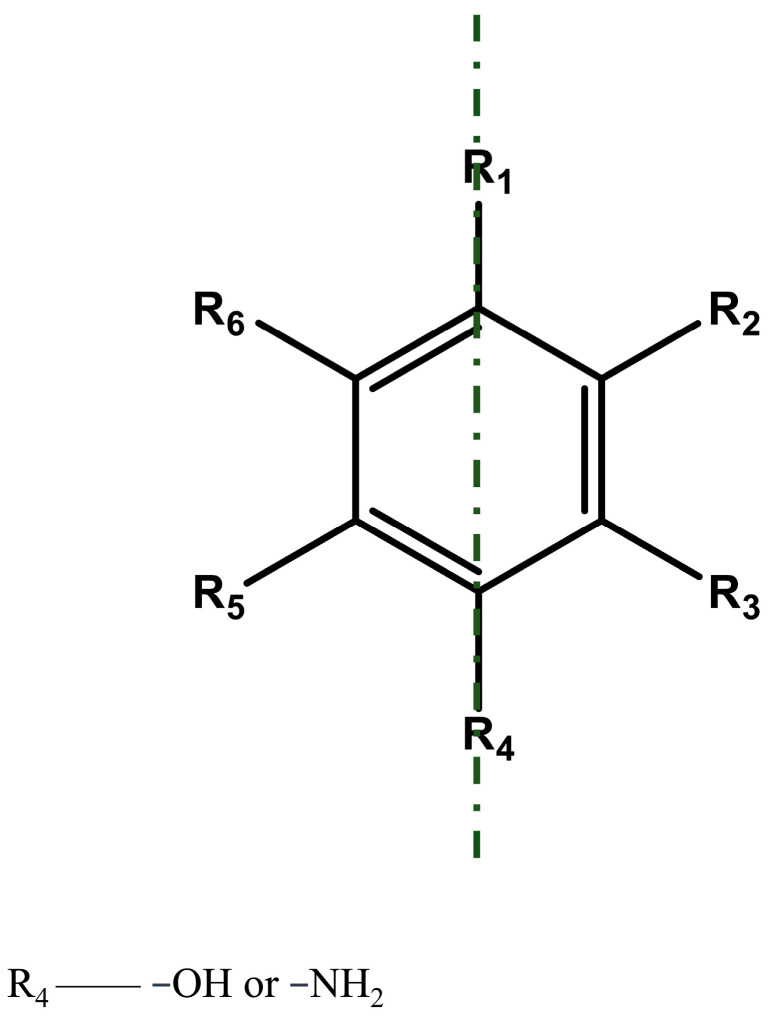
Structural characteristics might be *o*-hydroxylated by 4-hydroxyphenylacetate 3-hydroxylases.

To summarize, the aromatic compounds that can be hydroxylated by 4HPA3Hs should have the following three characteristics (Figure 6): (a) Contain at least one benzene ring; (b) the benzene ring has a hydroxyl or analog substituent at the R4 position; and (c) with R1 and R4 on the axis of symmetry, at least one side of the substituents (R2 and R3 or R5 and R6) is −H. A hydroxyl group can be introduced at the *o*-position (either R3 or R5) where the substituent is −H and is in proximity to R4.

## 4. Protein Engineering Strategies of 4-Hydroxyphenylacetate 3-Hydroxylases

**Figure 7:**
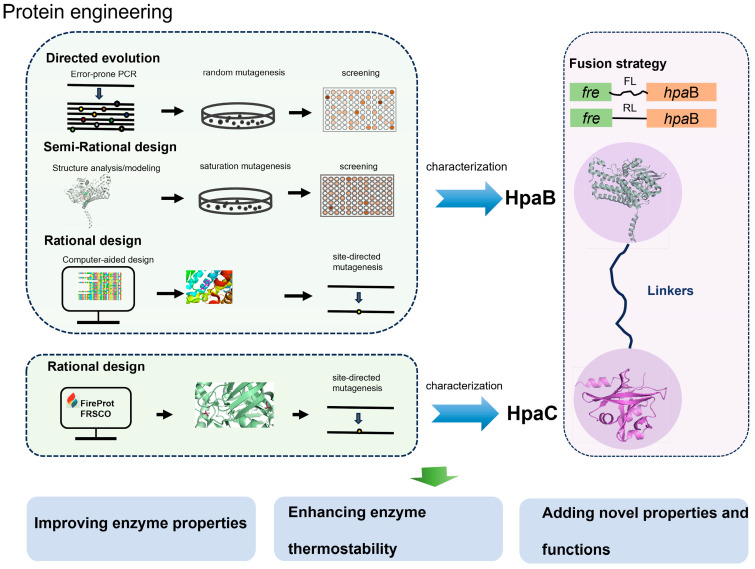
Protein engineering of 4-hydroxyphenylacetate 3-hydroxylases.

4HPA3Hs from various microorganisms have different catalytic properties and substrate spectra. Protein engineering is also used in 4HPA3Hs to broaden the substrate spectrum, enhance hydroxylation efficiency, and improve enzyme thermostability (Figure 7). Table 6 summarizes the *K_m_* and *K_cat_/K_m_* values of some mutants in the *o*-hydroxylation of phenolic derivatives. Table 7 presents a list of typical 4HPA3Hs that have been engineered using various protein engineering strategies.

Shen [34] et al. engineered β32-β33 loop (residues 207 to 217) in *Ec*HpaB and obtained the mutant XS6 with the highest affinity to bulky naringenin (*K_m_* =191.6 ± 33.6 μM), with the conversion rate increasing by 56.1% (1.89 mg/L/OD) (Table 6). In 2022, Herrmann [61] et al. achieved the identification and utilization of ferulic acid by single- or multi-point mutations at the Y301, S462, and M293 sites of *Ec*HpaB that could not oxidate ferulic acid originally.

In designing a pathway for the synthesis of hydroxytyrosol from tyrosine, Chen [50] et al. found that mutant H7 (S210T, A211M, and Q212G) showed dual tyrosine and tyramine hydroxylase functionality through saturation mutagenesis combined with the high-throughput screening of *Ec*HpaB variants. The titer of hydroxytyrosol was 1.89 g/L and the yield was 82%.

A structural analysis revealed that the introduction of M211 and G212 may have increased the flexibility of this ring, allowing the access of tyrosol or tyramine. In 2020, Yao [109] et al. chose a suitable 4HPA3H mutant (derived from *E. coli*) instead of tyrosol/tyramine hydroxylase to catalyze the most critical first step from tyrosine to L-DOPA by directed evolution. It removed the rate-limiting step in the entire catalytic route, and thus improved the conversion rate of tyrosine (>98%). In 2023, Zhang [63] et al. engineered *Ec*HpaB at the substrate pocket to improve the *o*-hydroxylation efficiency of resveratrol. Using structural analysis, the key residues I157 and A211 were chosen for further saturation mutagenesis among seven residues (I157, V158, N159, S210, A211, Q212, and S462), except for R113, Y117, and H155. The catalytic activity of the variants I157L, A211D, and I157L/A211D for resveratrol were enhanced by 1.84, 2.07, and 2.46 times compared to the wide type, respectively. Xu [64] et al. successfully achieved a remarkable yield of 3-hydroxyphloretin at 2.03 g/L using the mutant Q212G/F292A/Q377N through the modification of the loop and substrate pocket on *Pa*HpaB.

Dhammaraj [48] et al. proved that 4HPA3Hs from *A. baumannii*could catalyze 4-HPA and *p*-coumaric acid to generate the corresponding trihydric phenols. 4HPA3Hs from *A. baumannii* converted 4-HPA into 2-(3,4, 5-Trihydroxyphenyl) acetic acid (3,4,5-THPA) in 100 min. The mutant Y398S can effectively improve the stability, and the enzyme activity was maintained at 30 °C and 35 °C for 24 h and 15 h, respectively. The production rate of 3,4,5-THCA from *p*-coumaric acid was increased from 26% to 50% [35]. The mutant S146A catalyzed the *o*-hydroxylation of 4-aminophenylacetic acid to produce 3-hydroxy-4-aminophenylacetic acid more effectively [97]. In 2017, Chenprakhon [98] et al. found that the mutant R263D had a double substrate specificity, which can catalyze the hydroxylation of tyramine into dopamine (up to 57%). The mutant R263D/Y398D can catalyze the hydroxylation of octopamine to produce norepinephrine, whereas the wild-type *Ab*HpaBC displayed no activity.

Furthermore, several studies have also focused on the reductase components of 4HPA3Hs. Rational design assisted by FireProt and FRESCO was applied to improve the thermostability of the reductase component *Ab*HpaC to ensure the supply of reductase mutants. A166L and A58P showed improved thermostability and catalytic efficiency [58].

Fusion strategy has been applied to 4HPA3Hs. When fusing the N-terminal of HpaB from *Klebsiella pneumoniae* (*Kp*HpaB) with the C-terminal of Fre from *E. coli* using A flexible linker (FL: SGGSGGSGGSAG) or a rigid linker (RL: AEAAAKEAAAKA) in the engineered strain, the yield of intermediate caffeic acid was 7-fold and 9.1-fold higher than that of the control, respectively [62]. Moreover, the hydroxylation efficiency was increased by 8.1-fold when combined with cofactor engineering, which promoted the regeneration of the FADH_2_ cofactor.

It is evident that the application of protein engineering to 4HPA3Hs will contribute to imparting diverse characteristics to them in addition to the origins. Based on protein sequences and structures, insights into key catalytic residues and the active site environment are crucial for controlling the hydroxylation reactions of enzymes. It will undoubtedly facilitate future work on protein engineering or enzyme redesign on 4HPA3Hs, promoting the development of bioactivated methods for synthesizing valuable plant phenols.

**Figure 8:**
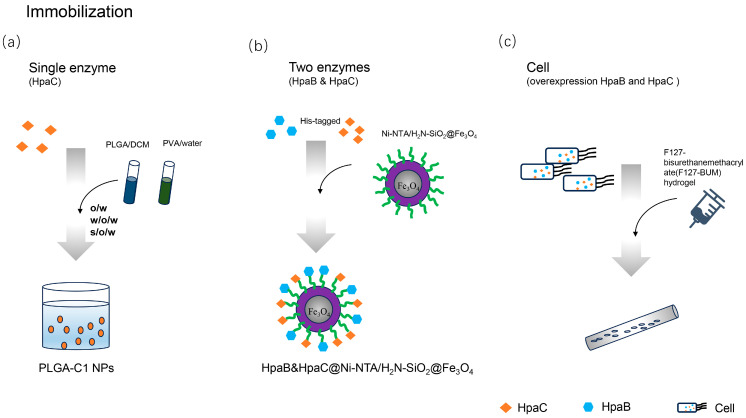
The illustration depicts the immobilized application of 4-hydroxyphenylacetate 3-hydroxylases, including (**a**) a single enzyme (HpaC), (**b**) two enzymes (HpaB and HpaC), and (**c**) cells (overexpressing HpaB and HpaC). Orange circle (subfigure **a**) represents the immobilized enzyme, green line (subfigure **b**) signifies the Ni-NTA, and tube (subfigure **c**) depicts the immobilized cells.

In addition, some immobilized applications of 4HPA3Hs have also been implemented in recent years (Figure 8). Sawasdee [110] adopted a double emulsion solvent evaporation method, encapsulating *Ab*HpaC(C1) with biodegradable poly(lactide-co-glycolide (PLGA) nanoparticles (NPs). PLGA-C1NPs prepared by solid in oil in water (s/o/w) enhanced the enzymatic activity, stability, and regeneration efficiency(Figure 8a). And they maintained 50% activity after 14 cycles of reuse. The co-immobilization of multiple enzymes also improved the stability and utilization of the multi-enzyme reaction system. Liao [111] et al. first used Ni2^+^-nitrilotriacetic acid-functionalized magnetic silica nanoparticles (Ni-NTA/H_2_N-SiO_2_@Fe_3_O_4_) to co-immobilize His-tagged *Ec*HpaB and *Ec*HpaC in 2020(Figure 8b). The separation, recovery, and aggregation of the two distinct enzymes in 4HPA3Hs were found to be straightforward, allowing for an improved catalytic efficiency due to the efficient transfer of reduced flavin between the enzymes. The activity of co-immobilized 4HPA3H was 2.6-times greater than that of the free enzyme. Additionally, the co-immobilization of two components of 4HPA3Hs improved the thermostability and alkali resistance. The activity was preserved at 76.6% after 12 days and the catalytic activity remained above 60% after seven cycles of reuse.

The hydroxylation of 4HPA3H in vitro requires a considerable amount of expensive NAD(P)H, which is coupled to a formate dehydrogenase for NAD(P)H supply [61]. An economical whole-cell catalysis approach has been employed, using the NAD(P)H and reduced flavins required for 4HPA3Hs for the cell’s inherent metabolism. Johnston [112] used F127-bisurethanemethacrylate(F127-BUM) hydrogel to immobilize an engineered strain assembled with 4HPA3Hs for producing L-DOPA(Figure 8c). They showed that the lyophilization of the cells did not affect the L-DOPA production activity, and the efficiency of the repeated production stage was better than that of the liquid culture. Forming immobilized bacterial consortia with yeast, the activity was not reduced after five cycles of reuse at 33.5 °C, and remained 100% efficient when rehydrated even after long-term storage at room temperature for 3 months.

In recent years, the hydroxylation efficiency of 4HPA3Hs has led to frequent employment in the construction of cell factories. 4HPA3H enzymes have the potential of industrial application, but there is little research on immobilization. More suitable immobilization materials and efficient processes still need to be further developed and improved.

## 5. Summary and Future Outlook

In recent years, the crystal structures of 4-hydroxyphenylacetate 3-hydroxylases have been elucidated, facilitating their extensive application in the hydroxylation reactions of numerous high-value natural products. 4HPA3Hs are multifunctional hydroxylases capable of o-hydroxylating a broad array of substrates, including numerous aromatic compounds with phenolic structures and even some alkaloid analogs. Owing to the meticulous investigation of 4HPA3Hs, many analogs hydroxylated by P450s, tyrosine monooxygenases, and other monooxygenases with intricate mechanisms can be further modified, at present, using native or engineered 4HPA3Hs. Additionally, the utilization of 4HPA3Hs circumvents potential challenges created by P450s, such as expression difficulties, costly cofactors, and suboptimal conversion rates, resulting in a low industrialization potential. At present, it has been suggested that 4HPA3Hs may be more suited for use in prokaryotic strains, as they displayed inefficient activity in yeasts [88,113,114]. 4HPA3Hs have been employed as direct substitutes for P450s in the de novo synthesis of plant phenolic derivatives due to the high catalytic activity of 4HPA3Hs. However, few studies on enhancing product yield have been conducted by engineering 4HPA3Hs while synthesizing certain phenolics.

Although 4HPA3Hs act on a wide range of substrates, their activity is limited to the o-hydroxylation of phenolic-structure analogs. Early research on 4HPA3Hs mainly focused on the properties and mechanisms of bifunctional enzymes. In recent years, 4HPA3Hs have been increasingly employed for the synthesis of plant products. However, 4HPA3Hs can react with multiple intermediates in the reaction process synchronously, resulting in the product not being ideal [50]. Therefore, it is important to apply strategies such as protein engineering to improve the substrate specificity. It has been reported that a fractional reaction using separately cultured cells can weaken the shunt of the end product [115]. However, there is a lack of effective strategies for the efficient and sustainable synthesis of plant polyphenols.

In addition, few studies have reported the correlation and difference of single/two-component monooxygenases. With the aid of protein engineering, 4HPA3Hs can replace certain P450s with complex mechanisms to perform the same or similar hydroxylation reactions. Nonetheless, there are research gaps regarding the differences and connections among enzymes that can catalyze the same reaction but have different mechanisms. Further research on 4HPA3Hs could offer novel insights into how to employ complex enzymes to catalyze specific reactions. This may also mean that the P450s required in complex product synthesis pathways can be functionally replaced by enzymes corresponding to alternative prokaryotic sources, facilitating the acquisition of natural products externally from plants.

4-Hydroxyphenylacetate 3-hydroxylases exhibit relatively a simple catalytic mechanism and high catalytic efficiency, and have great potential for enzyme engineering and application. In-depth research on 4HPA3Hs should be conducted using protein engineering, cofactor engineering, and rational/semi-rational approaches combined with machine learning, bioinformatics, and molecular biotechnology. This further reveals the potential applications of 4HPA3Hs, such as broadening their substrate spectrum and improving their catalytic efficiency and thermal stability. On the one hand, they can hydroxylate natural or artificial substrates that were originally incapable of being catalyzed after modification. On the other hand, applying the engineered 4HPA3Hs to chassis cell construction contributes to the de novo synthesis of natural products. Furthermore, the integration of targeted engineering and novel immobilized materials will enhance the industrial production of valuable natural products. These approaches will further reveal the considerable application potential of 4HPA3Hs in promoting a sustainable circular economy.

## Figures and Tables

**Table 1:** Reductase component and oxygenase component of 4HPA3Hs derived from various organisms [31].

Organism	Uniprot ID (Oxygenase Component)	Reductase Component	Oxygenase Component	References
*Acinetobacter baumannii*	Q6Q272	C1	C2	[7]
		(35.5 kDa)	(47 kDa)	
*Thermus thermophilus*	Q5SJP8	HpaC	HpaB	[32,33]
		(16.1 kDa)	(54.3 kDa)	
*Escherichia coli*	Q57160	HpaC	HpaB	[14,34,35]
		(17 kDa)	(59 kDa)	
		FAD	FAD	
*Pseudomonas aeruginosa*	Q9HWT7	HpaC	HpaB	[36]
		(19.4 kDa)	(58 kDa)	
*Geobacillus * sp.	Q4L1M7		HpaH	[37]
			(56.3 kDa)	
*Bacillus subtilis*	C0SPC0		yoaI	[38]
*Geobacillus thermodenitrificans*	A4IT51		GTNG_3160	[39]
*Sulfolobus tokodaii*		HpaC	HpaB	
		(18 kDa)	-	[40]
*Pseudomonas putida*		MhaB	MhaA	[41]
		(6 kDa)	(63 kDa)	
*Klebsiella Pneumoniae*	Q48440	HpaH	HpaA	[16,17]
		(19 kDa)	(59 kDa)	

**Table 2:** Crystal structure information of the oxygenase components of 4HPA3Hs.

Gene	Domains of the Oxygenase Component			Key Conserved Residues	UniProt ID	PDB ID	Reference
	N-Terminal α-Helix Domain	Intermediate Domain	C-Terminal Domain				
*Ab*HpaB	24–143	144–237	238–422	——	Q6Q272	2jbr, 2jbs, 2jbt	[44]
*Tt*HpaB	2–138	139–266	267–481	R100-Y104-H142	Q5SJP8	2yyg, 2yyi, 2yyj	[32]
*Ec*HpaB	12–148	155–281	296–489	R113-Y117-H155	Q57160	6qyh, 6qyi	[34,35]

**Table 3:** Crystal structure information of 4HPA3H reductase components.

Gene	Length (aa)	UniProt ID	PDB ID	Reference
*Ab*HpaC	315	Q6Q271	5ZYR (unpublished); 5ZC2	[52]
*Tt*HpaC	149	Q5SJP7	2ECR; 2ECU; 2ED4	[33]
*St*HpaC	156	Q974C9	2D36; 2D37; 2D38	[40]

**Table 4:** Kinetic parameters of various HpaBs in the *o*-hydroxylation of phenol derivatives.

Gene	Substrates	T (°C)	pH	*K_m_* (μM)	*K_cat_* (min^−1^)	*K_cat_/K_m_* (s^−1^mM^−1^)	Reference
*Ec*HpaB	4-HPA	RT	7.5	9.4 ± 1.6	264 ±12	470.000	[35]
	4-HPA	30	7.0	31 ± 4	283.2 ± 5.8	152.250	[59]
	4-HPA	30	7.0	18.4 ± 1.1	64.8 ± 1.0	58.6957	[60]
	DHPA	RT	7.5	46.1 ± 5.3	126 ± 5.4	46.000	[35]
	Hydroxymandelic acid	RT	7.5	24.7 ± 6.5	162 ± 12	110.000	[35]
	Tyrosol	RT	7.5	33.9 ± 7.0	90 ± 6	44.000	[35]
	*p*-Coumaric acid	RT	7.5	53.9 ± 16.6	36 ± 4.2	11.000	[35]
	*p*-Coumaric acid	30	7.0	137.6 ± 21.0	23.2 ± 0.7	2.800	[34]
	*p*-Coumaric acid	30	7.0	648.26 ± 111.82	8.95 ^a^	0.2308 ^a^	[62]
	phenol	RT	7.5	252.9 ± 54.6	18 ± 0.6	1.100	[35]
	Methyl hydroxybenzoate	RT	7.5	514.8 ± 103.0	12 ± 0.6	0.390	[35]
	Umbelliferone	30	7.0	217.0 ± 60.6	25.1 ± 2.4	1.900	[34]
	Umbelliferone	30	7.0	262.2 ± 40.3	4.0 ± 0.2	0.2543	
	Naringenin	30	7.0	349.8 ± 77.6	9.0 ± 0.3	0.400	[34]
	Naringenin	30	7.0	281 ± 68	0.25 ± 0.03	0.015	[59]
	Resveratrol	30	7.0	174.3 ± 17.9	26.2 ± 0.6	2.500	[34]
	Resveratrol	30	7.0	145.1 ± 19.2	5.6 ± 0.2	0.6432	[60]
	Resveratrol	30	7.4	670 ± 120	0.81 ± 0.057	0.020	[63]
*Ro*HpaB	4-HPA	30	7.0	170 ± 32	6.4 ± 0.4	0.627	[59]
	Naringenin	30	7.0	116 ± 4	1.68 ± 0.04	0.241	[59]
*Pp*HpaB	4-HPA	30	7.0	30 ± 5	287.3 ± 4.7	159.617	[59]
	Naringenin	30	7.0	79 ± 11	0.25 ± 0.01	0.053	[59]
*Pa*HpaB	Phloretin	30	7.4	0.213	0.781	0.061	[64]
*Kp*HpaB	4-HPA	30	7.0	26 ± 0.2	494.0 ± 0.5	316.667	[59]
	*p*-Coumaric acid	30	7.0	725.19 ± 6.82	2.22 ± 0.001 ^a^	0.05107± 0.00164	[62]
	Naringenin	30	7.0	364 ± 20	0.22 ± 0.01	0.010	[59]

^a^ Data were calculated based on the referenced literature.

**Table 5:** Monooxygenases capable of catalyzing the *o*-hydroxylation of aromatic compounds.

Enzyme	Origin	Substrate	Product	Yield ^a^	Yield ^b^ (4HPA3H)
*p*-Coumarate 3-hydroxylase (C3H)	*Arabidopsis*	*p*-Coumaric acid	Caffeic acid	7.2 mg/L [65]	18.74 g/L [66]
CYP199A2_F185L	*Rhodopseudomonas palustris*	*p*-Coumaric acid	Caffeic acid	2.8 g/L [67]	
Tyrosinase	mushroom	Tyrosol	Hydroxytyrosol	N/A ^c^ [68]	6.6 mM [36]
CYP84A1(F5H)	*Arabidopsis*	Ferulic acid	5-hydroxyferulic acid	N/A [69]	5.3 mM [36]
Tyrosinase	*Saccharothrix espanaensis* MA4680	Resveratrol	Piceatannol	N/A [70]	23 mM (5.2 g·L^−1^) [71]
Flavonoid 3′-hydroxylase (F3′H)	*Silybum marianum*	Naringenin	Eriodictyol	3.3 g/L [72]	14.10 mg/L [73]
CYP102A1 mutant M13	*Bacillus megaterium*	Naringenin	Eriodictyol	13.5 mg/L [74]	14.10 mg/L [73]
		Umbelliferone	Esculetin	337.10 μM (67.62%) [75]	N/A [61]
CYP102A1 mutant M10	*Bacillus megaterium*	phloretin	3-hydroxyphloretin	3.1 mM [76]	2.03 g/L [64]
Tyrosinase	*Mushroom*	Equol	3′-hydroxyequol	N/A [77]	1.06 g/L [78]

^a^ Reactions catalyzed by monooxygenases other than 4HPA3H. ^b^ Reaction catalyzed by 4HPA3H. ^c^ Not applicable.

**Table 6:** Kinetic parameters of the mutants of HpaBs in the *o*-hydroxylation of phenol derivatives.

Gene	Substrates	T (°C)	pH	*K_m_* (μM)	*K_cat_* (min^−1^)	*K_cat_/K_m_* (s^−1^mM^−1^)	Reference
*Ec*HpaB ^I157L^	Resveratrol	30	7.4	330 ± 56	0.77 ± 0.036	0.039	[63]
*Ec*HpaB ^A211D^	Resveratrol	30	7.4	600 ± 30	1.89 ± 0.040	0.053	
*Ec*HpaB ^I157L/A211D^	Resveratrol	30	7.4	1360 ± 300	7.79 ± 0.35	0.095	
XS2	*p*-Coumaric acid	30	7.0	387.9 ± 32.7	11.0 ± 0.4	0.470	[34]
	Umbelliferone	30	7.0	490.8 ± 17.3	16.9 ± 2.6	0.600	
	Resveratrol	30	7.0	404.6 ± 93.8	24.3 ± 2.4	1.000	
	Naringenin	30	7.0	1061.7 ± 21.1	6.5 ± 0.3	0.100	
XS3	*p*-Coumaric acid	30	7.0	235.6 ± 40.2	29.8 ± 1.3	2.100	
	Umbelliferone	30	7.0	266.2 ± 41.1	14.8 ± 0.6	0.900	
	Resveratrol	30	7.0	235.8 ± 52.8	33.9 ± 2.8	2.400	
	Naringenin	30	7.0	417.2 ± 10.2	9.0 ± 0.4	0.400	
XS4	*p*-Coumaric acid	30	7.0	210.8 ± 85.3	30.7 ± 3.7	2.400	
	Umbelliferone	30	7.0	204.5 ± 16.3	22.3 ± 0.5	1.800	
	Resveratrol	30	7.0	441.8 ± 22.1	50.8 ± 8.8	1.900	
	Naringenin	30	7.0	627.5 ± 75.0	6.1 ± 0.1	0.200	
XS5	*p*-Coumaric acid	30	7.0	235.3 ± 52.5	22.5 ± 1.3	1.560	
	Umbelliferone	30	7.0	346.4 ± 11.2	13.0 ± 1.6	0.600	
	Resveratrol	30	7.0	319.8 ± 9.9	20.0 ± 2.4	1.000	
	Naringenin	30	7.0	661.0 ± 93	7.8 ± 0.2	0.200	
XS6	*p*-Coumaric acid	30	7.0	132.1 ± 29.1	21.9 ± 1.0	2.800	
	Umbelliferone	30	7.0	176.9 ± 35.9	20.9 ± 1.4	2.000	
	Resveratrol	30	7.0	144.0 ± 22.9	25.0 ± 1.2	2.900	
	Naringenin	30	7.0	191.6 ± 33.6	9.0 ± 0.2	0.800	
*Pa*HpaB ^F292A^	Phloretin	30	7.4	0.150	0.922	0.102	[64]
*Pa*HpaB ^Q212G^	Phloretin	30	7.4	0.205	2.027	0.165	
*Pa*HpaB ^Q212G/Q376N^	Phloretin	30	7.4	0.523	2.520	0.080	
*Pa*HpaB ^Q212G/F292A^	Phloretin	30	7.4	0.124	1.368	0.184	
*Pa*HpaB ^Q212G/F292A/Q376N^	Phloretin	30	7.4	0.261	2.677	0.171	
*Ro*HpaB ^Y215A^	4-HPA	30	7.0	13 ± 5	22.03 ± 2.85	27.380	[59]
	Naringenin	30	7.0	3 ± 1	1.29 ± 0.03	6.361	
	Apigenin	30	7.0	27 ± 1	0.025 ± 0.0002	0.016	
	Kaempferol	30	7.0	519 ± 105	0.33 ± 0.04	0.011	

**Table 7:** Typically engineered 4HPA3Hs using various protein engineering strategies.

Gene	Strategies	Engineered Enzymes	Advantages	Reference
Oxygenase component				
*Ec*HpaB	Rational design	XS6	56.1% increased conversion (1.89 mg/L/OD)	[34]
*Ec*HpaB	Rational design	Y301, S462 and M293	Expanded substrate range (e.g., ferulic acid)	[61]
*Ec*HpaB	Semi-rational design	H7 (S210T, A211M, Q212G)	Dual functionality (tyrosine and tyramine hydroxylase); 17-fold higher activity on tyrosol; 271-fold higher on tyramine	[50]
*Ec*HpaB	Directed evolution	23F9-M4 (T15P, S210F, A211K, Q212F, D284E)	15-fold increase in L-DOPA yield	[109]
*Ec*HpaB	Semi-rational design	I157L, A211D, and I157L/A211D	1.94-, 2.6-, and 4.7-fold increase in catalytic efficiency (*K_cat_/K_m_*-resveratrol); 1.84-, 2.07-, and 2.46-times increase in catalytic activity for resveratrol	[63]
*Pa*HpaB	Rational design	Q212G/F292A/Q376N	3-hydroxyphloretin yield increased to 2.03 g/L	[64]
*Ab*HpaB	Rational engineering	Y398S	Stability improved (30 °C: 24 h, 35 °C: 15 h); 3,4,5-THCA yield increased from 26% to 50%	[48]
*Ab*HpaB	Rational design	S146A	Improved catalytic efficiency	[97]
*Ab*HpaB	Rational design	R263D	Double substrate specificity: 57% tyramine and 86% 4-HPA	[98]
		R263D/Y398D	Expanded substrate range (e.g., octopamine)	
Reductase component				
*Ab*HpaC	Rational design	A58P and A166L	Improvements in thermostability (T_m_ increased by 3–5 °C) and catalytic efficiency	[58]
4HPA3H				
*Ec*HpaC(Fre) and *Kp*HpaB	Fusion strategy	Fre-FL-*Kp*HpaBC and Fre-RL-*Kp*HpaBC	7- and 9.1-fold increase in caffeic acid yield	[62]

## Data Availability

Not applicable as this is a review article and does not generate or analyze any new data.
